# Red Wine and Sexual Function in Men: An Original Point of View

**DOI:** 10.3390/jcm12123883

**Published:** 2023-06-07

**Authors:** Livia Basile, Rosita A. Condorelli, Aldo E. Calogero, Rossella Cannarella, Federica Barbagallo, Andrea Crafa, Antonio Aversa, Sandro La Vignera

**Affiliations:** 1 Department of Clinical and Experimental Medicine, University of Catania, 95125 Catania, Italy; livia.basile@unict.it (L.B.); rosita.condorelli@unict.it (R.A.C.); acaloger@unict.it (A.E.C.); rossella.cannarella@phd.unict.it (R.C.); federica.barbagallo11@gmail.com (F.B.); andrea.crafa@outlook.it (A.C.); 2 Department of Experimental and Clinical Medicine, University Magna Graecia of Catanzaro, 88100 Catanzaro, Italy; aversa@unicz.it

**Keywords:** red wine, sexual function, erectile dysfunction

## Abstract

Red wine is a rich source of nutrients whose biological properties have inspired numerous scientific studies. Indeed, it has been widely reported that there is a correlation between the positive health effects of moderate consumption of red wine and its phenolic content, which, due to its antioxidant activity, has proved to be useful in the improvement of various diseases, such as cardiovascular diseases, metabolic syndrome, cognitive disorders, depression, and cancer. It is a common opinion that the antioxidant activity of red wine is to be ascribed to its entire content of polyphenols, which act synergistically and not as a single component. Furthermore, this health-promoting effect of red wine can also be linked to its ethanol content, which has shown a wide array of biological properties. Beyond this evidence, very little is known about a possible correlation between moderate consumption of red wine and male sexual function. This brief review aimed to evaluate the effects of moderate consumption of red wine on erectile function. To accomplish this, Pubmed and Google Scholar databases were searched to retrieve the most relevant studies on this topic. The evidence so far collected has shown that red wine, if consumed in moderation, can be potentially beneficial for patients with erectile dysfunction as well as can positively influence reproductive function through mechanisms that depend on the vasorelaxant properties of red wine and its antioxidant properties.

## 1. Introduction

Red wine is an alcoholic beverage obtained from the fermentation of dark-colored grapes that are rich in phytonutrients to which the organoleptic properties and beneficial health effects on the health of red wine have been attributed [1]. Chemically red wine is a complex mixture of organic compounds, including water, ethanol (EtOH), glycerol, sugars, vitamins, organic acids, aldehydes, ketones, esters, minerals, lipids, and phenolics [2]. The beneficial health effects of red wine consumption have been mainly associated with its polyphenolic content, whose total amount ranges between 2000 and 6000 mg/L [3] and consists of flavonoids and non-flavonoids. Flavonoids include flavonols (quercetin and myricetin), flavanols (catechin and epicatechin), and anthocyanins. Stilbenes (resveratrol), hydroxycinnamates (caftaric, caffeic, and coutaric acids), and hydroxybenzoates belong to the class of non-flavonoid compounds [4].

While it was widely accepted that moderate intake of red wine induces positive health effects [5], the real properties of its components on cellular systems and the mechanism underlying their molecular interactions with organic systems remain to be elucidated. A major issue concerns the alcohol content of red wine and the threshold between the amount of alcohol that can adversely affect biological functions and the amount of alcohol that can have beneficial effects on human health [6]. It was observed that EtOH present in red wine produces a broad spectrum of biological activities, such as influencing the composition of cholesterol and the activity of its metabolizing enzymes, which include the cytochrome p450 family, the glutathione S-transferase superfamily, and N-acetyl transferases [7]. Moderate alcohol consumption (15–30 g/day) has been associated with a cardioprotective effect due to an increase in plasma levels of high-density lipoprotein (HDL), cholesterol, and a reduction in platelet adhesiveness, all responsible for atherosclerosis [8]. As shown by meta-analysis studies, the daily intake of 1 to 3 glasses of standard drinks containing about 10–30 g of alcohol reduced cardiovascular risk [9,10,11]. To the same extent, the pro-oxidant effect [12,13] exerted by EtOH seems to be neutralized by the high content of polyphenol in red wine, which exhibits clear antioxidant activity [14]. Chemical analysis of red wine has revealed that it contains 10 times more polyphenols than other types of wine due to fermentation of darker red or black grapes, including skins and seeds [15].

It is commonly believed that the antioxidant activity of red wine can be attributed to its entire polyphenol content [16]. It can therefore be hypothesized that the total antioxidant capacity of red wine may not depend on a single compound but rather results from the synergistic antioxidant effect of other phytochemicals present in it [17]. Polyphenols show also an anti-inflammatory activity that derives from two different functions: the radical scavenging properties that can block oxidative stress signaling or the modulation of pro-inflammatory signaling transduction [18]. These findings underlie the concept known as the “French paradox”, which reported a low prevalence of coronary heart disease in France in both male and female populations despite eating a diet characterized by a high content of saturated fat [19]. In fact, the authors of this study ascribed this discrepancy to the free radical scavenging activity of polyphenolic compounds contained in red wine. Since then, much attention has been paid to the French situation, inspiring further studies on various aspects of the properties of red wine [20]. Much more is currently known about the phenolic composition of red wine, and several in vitro experiments have been carried out to support findings on the biological properties of red wine flavonoids [21,22,23]. Improvement of clinical outcomes of several diseases, such as cardiovascular disease, metabolic syndrome, cognitive disorders, depression, and cancer, has been mainly attributed to the abundance of antioxidants [24,25]. These include resveratrol, anthocyanins, catechins, and flavonoids in red wine, which can counteract the action of a wide array of oxidant molecules, such as reactive oxygen species (ROS), nitrogen oxide, and chlorine, and also suppress the synthesis of these reactive species. The consumption of red wine has also been seen as a contributing factor in improving blood pressure outcomes in hypertensive patients [25]. Evidence from epidemiological studies [26,27,28] has shown that moderate daily consumption (20–30 g) of red wine could positively influence cardiovascular health and minimize the risk related to all-cause mortality among middle-aged adults and elderly people. Although numerous pieces of evidence exist on the cardiovascular protection associated with moderate consumption of red wine [29,30,31,32,33], there are still few studies on the possible effect of red wine on male gonadal function.

In this brief review, the effects of moderate consumption of red wine (as a complex mixture of ethanol and phytocompounds) on erectile function were evaluated. Pubmed and Google Scholar were searched using the following keywords: “red wine and sexual function”, “red wine and sexual desire”, “red wine and male infertility”, and “red wine and spermatozoa” to find the most relevant studies that indicate a correlation between regular red wine intake and sexual function. Table 1 reports studies performed in vitro, in vivo, and in humans. These later were described in Table 2.

## 2. Red Wine and Gonadal Function

Regular intake of different types of red wine appears to affect serum follicle-stimulating hormone (FSH), testosterone, 17β-estradiol, and prolactin serum levels in young adult male rats, playing a role in modulating reproductive outcomes. These effects appear to be dose-dependent and influenced by wine characteristics [14]. In the same way, the beneficial effect of red wine on the vascular system could also be supposed for erectile dysfunction (ED), currently considered the first manifestation of atherosclerosis and therefore a marker of systemic vascular disease rather than an age-related complication of heart disease [51]. However, in this context, the role of alcohol and polyphenol content in red wine remains still to be clarified [52,53].

## 3. Red Wine Polyphenols and Erectile Dysfunction

Penile erection is a neuro-vascular event characterized by smooth muscle relaxation and increased blood flow to the penile cavernous level leading to a veno-occlusive mechanism [42]. It is widely recognized that nitric oxide (NO) increases cGMP production by cavernous smooth muscle cells, which, through activation of protein kinase G, causes increased calcium efflux with smooth muscle relaxation. In ED, the small vessels of the penis undergo structural and functional changes in the endothelium [46], resulting in decreased NO bioavailability as a consequence of a reduced synthesis or increased NO degradation in perivascular smooth muscle [54]. Red wine polyphenols are thought to act directly through the modulation of the NO system [34], increasing the production of endothelial NO synthase and reducing the synthesis of endothelin-1, a potent peptide with vasoconstrictor activity, the overexpression of which is considered a fundamental factor in the development of vascular disease and atherosclerosis [55]. NO from neuronal and endothelial cells in the corpora cavernosa (CC) of the penis actives soluble guanylyl cyclase (sGC), which catalyzes the conversion of guanosine triphosphate to cyclic guanosine monophosphate (cGMP). Intracellular cyclic GMP is a biochemical mediator that interferes with the activity of calcium channels, providing a reduction of the amounts of cytosolic calcium and regulating the activity of contractile proteins. This dual action allows for relaxation of the smooth muscle of the CC, causing penile vasodilation and therefore its erection [56].

In in vitro experiments, red wine polyphenols exhibited relaxant effects in the arteries of different vascular beds mainly via a NO/sGC-dependent mechanism [35,36]. However, there is some controversy regarding the possible involvement of the NO system in achieving the relaxing activity of red wine and whether this effect could result from a combined action of polyphenols and alcohol contained in it. The in vitro study by Boydens and colleagues evaluated the relaxant capacity of two red wine polyphenols on isolated mouse aorta and CCs along with their contribution to oxidative stress-induced ED [37]. From the results obtained, it was hypothesized that two polyphenols, quercetin, and resveratrol act as potent vasodilators of the mouse aorta. However, the relaxant effect observed in the CC was probably due to resveratrol alone, suggesting that the experimental results are not in line with a NO/sGC-dependent mechanism. According to the author, this disparity in the literature data could be ascribed to species differences and different experimental models. Alternatively, given the structural diversity between quercetin and resveratrol, the authors hypothesized that quercetin could act through a target-specific activation or that its receptor could have a higher affinity/selectivity for the mouse aorta than the CC. Furthermore, in the latter, resveratrol showed a greater antioxidant activity than quercetin, manifesting a pronounced reduction in neuronal NO responses. Endothelial NO reduces cardiovascular risk through vasoprotective effects in the development of atherosclerosis [57].

Polyphenols have antiatherosclerotic properties [58] also by reducing the expression of adhesion molecules and growth factors responsible for the migration and proliferation of vascular smooth muscle cells. To the same extent, EtOH present in varying amounts in wine has been shown to exert antiatherogenic effects [59]. Data from animal and human studies attributed to EtOH antithrombotic effects and the ability to increase high-density lipoprotein levels [60,61]. In this context, this may explain the results of epidemiological studies reporting a relationship between moderate consumption of red wine and a reduced risk of coronary heart disease [33,62,63]. The effects of chronic red wine intake on the expression of angiogenetic factors, such as the vascular endothelial growth factor (VEGF), angiopoietin 1, angiopoietin 2, and its receptors in rat erectile tissue were studied [38]. In fact, NO seems to play an important role in the development of ED thanks to its involvement in the angiogenic process. Based on mounting evidence, NO regulates VEGF-induced angiogenesis [64]. Results from experimental rat models of ED have revealed that intracavernous administration of VEGF can have positive effects on erectile function [43,44]. Angiogenic factors angiopoietin1 (Ang1) and angiopoietin2 (Ang2) crosstalk with VEGF to modulate angiogenesis. Both Ang1 and Ang2 act on vascular development and maturation by interacting with endothelial-specific tyrosine kinase 2 with immunoglobulin-like and epidermal growth factor homology domains (Tie2). The results from Neves’ study showed that red wine-treated rats had reduced VEGF expression, which is consistent with a decrease in the angiogenesis process in the CC of these animals. This effect could be attributed to the antiatherosclerotic properties of red wine on plaque formation. However, unlike the EtOH group, the obtained data revealed an increase in the expression of Ang1 and Ang2 at CC levels in the red wine group, which may balance the loss of VEGF. Thus, it is likely that this protective effect on the vascular system can be ascribed to the antioxidants present in the red wine that interfere with angiopoietin/Tie2-dependent mechanisms for maintaining cavernous tissue vascularization.

Yetik-Anacak and coworkers [39] suggested an alternative hypothesis that provided an underlying rationale for the protective effect of red wine on ED. In this study, resveratrol is believed to relax the CC in mice by inducing hydrogen sulfide (H_2_S) formation in a NO pathway-independent manner. H_2_S is an endogenous gas transmitter endowed with vasorelaxant and pro-erectile activity, produced in the CC in response to neural excitation from L-cysteine through the intervention of two enzymes: cystathionine-gamma-lyase (CSE) and cystathionine-β-synthase (CBS) [65]. Although crosstalk between the two gas transmitters NO and H_2_S in angiogenesis and vascular relaxation can be hypothesized, in this study, the effect of resveratrol on H_2_S production in penile tissues was consistent with a direct action on CBS and/or CSE. The data obtained, in fact, showed that resveratrol significantly increased the relaxation induced by L-cysteine, and H2S inhibitors inhibited this effect.

## 4. Red Wine Polyphenols and Gonad-Related Hormones

The antioxidant properties of polyphenols found in red wine appear to be beneficial for the male reproductive system, suggesting a positive correlation between red wine consumption and testosterone serum levels. Altered physiological hormone levels in the blood, including lower testosterone or testosterone/luteinizing hormone ratio, higher 17β-estradiol levels, or hyperprolactinemia, may adversely affect male fertility. As demonstrated by an in vitro study, red wine has been found to increase testosterone levels by suppressing the activity of the aromatase enzyme CYP19A1 responsible for its conversion to 17β-estradiol [40]. Furthermore, red wine has been proven to block testosterone metabolism through glucuronidation, which inhibits its excretion [41]. A study by Oczkowski and colleagues explored the effects of regular consumption of different red wines on hormonal reproductive profiles and total antioxidant status in rats [14]. Dry red wine (D-RW) showed the highest content of phenolic compounds and the highest antioxidant activity in comparison to semi-sweet (SD-RW), sweet (S-RW), and semi-sweet (SS-RW) red wines considered in this analysis. Furthermore, the investigators reported that the intake of red wine did not alter the serum hormone levels in rats, and the antioxidant status showed no difference even compared to the control group. These observations led the authors to hypothesize that the pro-oxidative effects of wine EtOH on the reproductive system could be partly reduced by the antioxidant activity of phenolic compounds naturally present in red wines. Furthermore, the hypothesis of a threshold relationship between the consumption of red wine, intended as a fraction of EtOH consumed, and hormones measured could explain why in the D-RW, SD-RW, and SS-RW groups, where consumption was low, reproductive hormone levels did not change compared to the control groups. In contrast, the differences in the analyzed hormones (FSH, 17β-estradiol, and prolactin) were observed in the S-RW group characterized by a higher intake of wine than the other groups due to the presence of a greater quantity of sugar. These data are supported by evidence that in humans and rats, chronic intake of EtOH increases prolactin levels and aromatase expression in the rat adipose tissue chronically exposed to red wine or alcohol. Furthermore, the authors attributed these high serum estrogen levels in male animals chronically treated with red wine to their phytoestrogen content and/or the absence of their excretion by the liver. However, higher FSH levels in rats consuming S-RW compared to controls and D-RW could be a consequence of the highest EtOH consumption in this group. In this study, despite the higher consumption of red wine and alcohol in the S-RW group, testosterone levels did not decrease, probably due to the protective effect of caffeic acid, a polyphenol present in large quantities in this type of wine. Moreover, rats given dry red wine showed very low testosterone levels, although their alcohol intake was the lowest, and this wine was characterized by the highest amount of phenols. According to the authors, phytoestrogens may influence steroidogenesis, particularly in dry red wine. A report on the effect of regular red wine consumption on testicular profiles in male rats revealed increased perfusion of the testicular tissue without morphological differences in the seminiferous tubules containing germ cells at different stages of development [45].

## 5. Red Wine and Female Sexual Function

In addition to investigating a positive correlation between moderate red wine consumption and sexual health in men, Mondaini and colleagues also investigated the potential role of red wine intake in women’s sexual function [47]. As in men, endothelial dysfunction is believed to be part of the pathophysiology of female sexual dysfunction, although this relationship needs to be further elucidated. The NO pathway has also been recognized as crucial for female sexuality, although the female sexual response cycle appears to encompass factors different from that of men, such as psychological factors that include motivation and availability to initiate sex. In this study, the authors evaluated the effects of red wine assumption on several organic sexual responses, such as vulvar swelling, vaginal lubrication, and engorgement-inducing endothelium-dependent dilation of blood vessels. A total of 798 women living in the Chianti area (Tuscany, Italy) were enrolled in this study and were asked to complete the Female Sexual Function Index (FSFI) questionnaire. Fascinatingly, women consuming moderate (one–two glasses a day) red wine (group 1) showed higher FSFI scores for sexual desire and lubrication with an overall improvement in sexual function compared to teetotaler women (group 2) or “occasional drinkers” who consumed less than one glass per day of any type of wine or other alcoholic beverages. Considering that age usually correlates inversely with sexual function, these findings appear to be very interesting, as the women in group 1 were older than in groups 2 and 3. According to the authors, these findings could be due to a synergistic effect of both polyphenols and alcohol content in red wine. In this study, red wine intake resulted in an overall improvement of sexual function in group 1 compared to group 3, which was composed of occasional drinkers of other types of alcoholic beverages, including white wine and to a lesser extent red wine. Red wine polyphenols, through an improvement of NO-dependent endothelial function, provide a rationale for the improvement of peripheral arterial vasodilatory properties. This improvement translates into the positive effects of red wine on metabolic syndrome and arterial-mediated phases of female sexual function. Thus, the NO/cGMP pathway appears to be a key factor exerting local vasodilatory activity of female sexual organs, such as the vagina and clitoris, a sine qua non for an adequate female sexual response [66].

## 6. Effects of Alcohol

**Figure 1:**
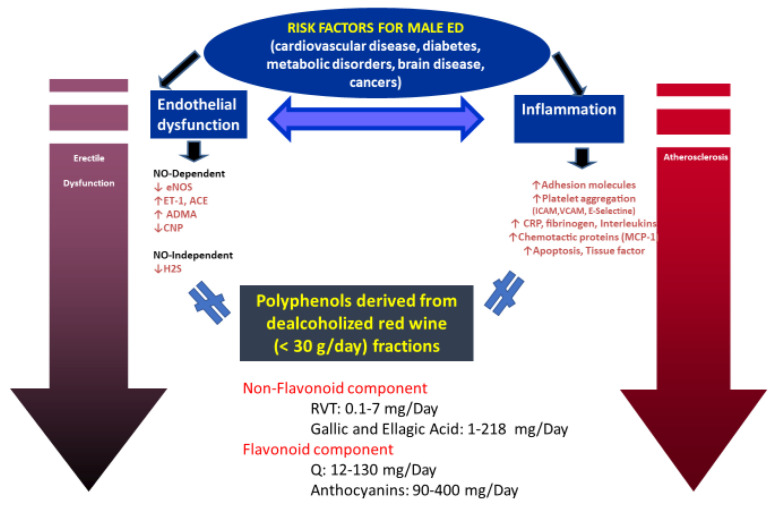
Red wine (intact or dealcoholized) contains high concentrations of polyphenolic compounds, such as flavonoids (catechin, epicatechin, quercetin, anthocyanins, and procyanidins), resveratrol [3,5,40–trihydroxystilbene (RVT)], and polymeric tannins. RVT and quercetin (Q) may protect against male erectile dysfunction and may induce penile smooth muscle relaxation (corpus cavernosum) through nitric oxide (NO)-dependent (eNOS-endothelin1 reduction) and independent (hydrogen dysulphide-H_2_S) mechanisms. Furthermore, the anti-inflammatory effects of red wine also protect against platelet aggregation and thus the progression of atherosclerosis (adapted from reference [29]).

Although moderate consumption of alcohol has been shown to have a protective effect on ED [48], possibly due to the long-term benefits of alcohol on increasing NO levels [67], alcohol affects sexual health in a different manner depending on the frequency and amount of consumption. While the consumption of alcohol in low doses promotes sexual behavior and desire, in higher doses, it is detrimental to sexuality. At the central level, alcohol acts as a depressant, reducing brain function, respiration, and circulation [68]. This depressive effect of alcohol appears to be related to the development of sexual dysfunction, mainly ED in men and lack of vaginal lubrication in women [67]. Chronic alcohol intake reduces testosterone levels, impairs spermatogenesis [69], and decreases testicular volume as well as can lead to feminization with increased ED severity, testicular atrophy, and gynecomastia [70]. Furthermore, in women, high alcohol consumption is harmful to sexual health. Indeed, it was associated with low sexual desire, anorgasmia, and dissatisfaction with one’s sexual functions [49]. In recent times, given the widespread awareness of the negative effects produced by alcohol abuse, there has been a growing interest in dealcoholized drinks. The dealcoholization of wine consists of the partial or total removal of alcohol using various methods. Membrane separation and thermal distillation are the most commonly used methods for commercial production [71]. The elimination of ethanol affects the quality of the wine but maintains its beneficial effects on health [50] due to the presence of phytonutrients as well as not causing negative effects related to the alcohol content. Furthermore, a greater number of people, including youngsters, pregnant women, drivers, and teetotalers, can consume dealcoholized wine (Figure 1).

## 7. Limitations of the Study and Caution in Data Interpretation

The effects of alcohol use are mainly harmful (based on daily consumption) on different organs and systems (diabetes, hypertension, liver, heart, etc.). This aspect is beyond the scope of this article. To this aspect, it should be added that there is insufficient evidence to support that antioxidant therapy improves sexual function in clinical practice, for example, only in the study by Shirai et al. Resveratrol (300 mg/d) within a combination with testofen, 600 mg/d; L-citrullines, 800 mg/d; and caffeine, 40 mg/d was evaluated by applying the IIEF (International Index of Erectile Function) for the evaluation of efficacy on male sexual function with good results [72]. The practical clinical message regarding the improvement of erectile dysfunction does not concern the possibility of improving sexual function with the consumption of red wines but only underlines the potential aspects of interest of this nutritional habit on sexual and reproductive health, which appears to be a field of interest for future clinical trials. Finally, the aspect relating to the usefulness of mobile applications regarding the most appropriate nutritional choices for male reproductive health also appears to be little explored and worthy of further investigation through well-conducted clinical studies [73].

## 8. Concluding Remarks

In conclusion, it is commonly believed that moderate consumption of red wine plays a valuable role in human health due to the co-presence of flavonoids and EtOH. Although many studies have explored the beneficial effects of red wine through in vitro and in vivo experimental models, there is a paucity of clinical trials supporting a relationship between moderate red wine consumption and male fertility. Based on the literature, it can be concluded that the antioxidant properties of polyphenols present in red wine appear to be beneficial for the reproductive system. Furthermore, the data reveal that the polyphenolic fraction in red wine can counteract the effects of EtOH or act directly on male reproduction. The effects of red wine intake on the hormonal regulation of the male reproductive system appear to depend on the type and amount of red wine. However, further investigations are necessary to confirm the precise role of both EtOH and polyphenols in the field of male and female sexual response, as well as to elucidate the specific mechanisms of their action.

## Figures and Tables

**Table 1:** Studies performed in vitro, in vivo, and in humans.

In Vitro	In Vivo	In Humans
Tekos, F. et al., 2021 Metabolites [16] Kaur, G. et al., 2007 J Thromb Haemost. [21] Tedesco, I. et al., 2021 Antioxidants [22] Shafreen, R.M.B. et al., 2021 Molecules [23] Cavallini, G. et al., 2016 J Nutr Health Aging [25] Duluc, L. et al., 2013 The International Journal of Biochemistry & Cell Biology [34] Chen, C.K. et al., 1996 Gen Pharmacol. [35] Shen, M. et al., 2013 Vascul Pharmacol. [36] Boydens, C. et al., 2014 J Sex Med. [37] Neves, D.R.G.L.M. et al., 2010 J Food Sci. [38] Yetik-Anacak, G. et al., 2015 J Sex Med. [39] Monteiro, R. et al., 2006 J Agric Food Chem. [40] Jenkinson, C. et al., 2012 Nutr. J. [41]	Oczkowski, M. et al., 2014 Food Funct. [14] De Paula, G.C. et al., 2021 Nutr Neurosci. [31] Zhang, Q. et al., 2010 Int J Androl. [42] Gholami, S.S. et al., 2003 J Urol [43] Kwangsung, P. et al., 2004 Eur Urol. [44] Isaac, U.E. et al., 2021 J Clin Med Kaz [45]	Bell, J. et al., 2000 Am J Clin Nutr. [8] Nigdikar, S.V. et al., 1998 Am J Clin Nutr. [13] Chiva-Blanch, G. et al., 2011 Am. J. Clin. Nutr. [18] Torres, A. et al., 2015 Revista Clínica Española [27] Nova, E. et al., 2019. Nutr Res. [28] Kaya, C. et al., 2006 Int J Impot Res. [46] Mondaini, N. et al., 2009 J Sex Med. [47] Chew, K.K. et al., 2009 J Sex Med. 2009 [48] Anil, K.B. et al., 2017 Asian J Psychiatr. [49] Chiva-Blanch, G. et al., 2012 Circulation Research [50]

**Table 2:** Studies in humans.

	Participants	Dose/Methods	Effects
ell, J. et al., 2000 Am J Clin Nutr. [8]	9 (5 men, 4 women)	120 mL/d of dealcoholized red wine reconstituted with either water or water and alcohol.	Moderate consumption of both red wine reconstituted with either water or water and alcohol induces an increase in the blood of (+)-catechin levels.
Nigdikar, S.V. et al., 1998 Am J Clin Nutr. [13]	30 men	375 mL/d of red wine or white wine; 1 g/d of red wine polyphenols in capsules, 1 g/d red wine polyphenols dissolved in white wine, or 400 mL/d alcoholic drink as vodka and lemonade (containing no polyphenols).	Red wine consumption provides beneficial effect on LDL oxidation.
Chiva-Blanch, G. et al., 2011 Am. J. Clin. Nutr. [18]	67 men	272 mL/d of red wine or dealcoholized red wine or 100 mL/d of gin.	Phenolic compounds in red wine interfere with leukocyte adhesion molecules. Both portions of ethanol and polyphenols in red wine modulate soluble inflammatory mediators.
Torres, A. et al., 2015 Revista Clínica Española [27]	16 (8 men, 8 women)	16 g/m2 of alcohol or different beverages (red wine, vodka, brandy, or rum).	Moderate red wine consumption improves both pro-inflammatory factors and serum antioxidant capacity (decreased in mean concentrations of hsCRP, TNFα, and IL-6) after a pro-atherogenic meal.
Nova, E. et al., 2019. Nutr Res. [28]	143 (56 men, 87 women)	Less than 4 alcoholic drinks per month (abstainers and occasional consumers); beer more than 80% of total alcohol intake (beer consumers); and wine, beer, and liquor (mixed beverage consumers). Total alcohol intake (g/d) was calculated as average grams of alcohol content per 100 mL of each alcoholic beverage.	Moderate red wine intake decreases pro-inflammatory factors and increased total antioxidant capacity (higher levels of HDL-c and adiponectin).
Kaya, C. et al., 2006 Int J Impot Res. [46]	57 men	Administration of the Sexual Health Inventory for Men (SHIM) 5-item questionnaire based on the International Index of Erectile Function (IIEF) questionnaire. Physical examination and analysis of fasting serum glucose and triglyceride, HDL cholesterol, and LDL cholesterol levels.	Reduction of NO bioavailability in patients with ED is related to structural and functional endothelial changes in small vessels of the penis.
Mondaini, N. et al., 2009 J Sex Med. [47]	798 women	One to two glasses of red wine (wine consumers) or less than one glass per day (occasional drinkers of wine or other alcoholic beverages).	Regular, moderate red wine improves sexual functioning in women (sexual desire and lubrication with an overall improvement in sexual function).
Chew, K.K. et al., 2009 J Sex Med. 2009 [48]	1580 men	Less than 1, 1 to 20, or more than 20 standard drinks a week, or less than 1, 1–5, or more than 5 days each week (current drinkers). Five or more standard drinks on one, or more days each week (binge drinkers). More than 20 standard drinks a week, or more than 4 standard drinks a day on 1 day, or more a week (high-risk drinkers). A standard drink consists of 375 mL of beer (3–4%), 100 mL of glass of wine (10–14%), or 30 mL nip of spirits (37–43%).	Moderate consumption of alcohol has a protective effect on ED.
Anil, K.B. et al., 2017 Asian J Psychiatr. [49]	80 women	90 to 359 mL/d of alcohol or 360 mL/d and above.	Alcohol dependence causes sexual dysfunction (low sex desire and orgasm-related problems.).
Chiva-Blanch, G. et al., 2012 Circulation Research [50]	67 men	100 mL/d of gin (30 g ethanol/day), 272 mL/d of red wine (30 g ethanol/day), or 272 mL/d of dealcoholized red wine.	Dealcoholized red wine decreases systolic and diastolic blood pressure via a NO-dependent mechanism.

## Data Availability

Not applicable.
